# Complementary and alternative medicine use in oncology: A questionnaire survey of patients and health care professionals

**DOI:** 10.1186/1471-2407-11-196

**Published:** 2011-05-24

**Authors:** Kah Hoong Chang, Rachel Brodie, Mei Ann Choong, Karl J Sweeney, Michael J Kerin

**Affiliations:** 1 Department of Surgery, University College Hospital Galway, National University of Ireland, Galway, Ireland

## Abstract

**Background:**

We aimed to investigate the prevalence and predictors of Complementary and Alternative Medicine (CAM) use among cancer patients and non-cancer volunteers, and to assess the knowledge of and attitudes toward CAM use in oncology among health care professionals.

**Methods:**

This is a cross-sectional questionnaire survey conducted in a single institution in Ireland. Survey was performed in outpatient and inpatient settings involving cancer patients and non-cancer volunteers. Clinicians and allied health care professionals were asked to complete a different questionnaire.

**Results:**

In 676 participants including 219 cancer patients; 301 non-cancer volunteers and 156 health care professionals, the overall prevalence of CAM use was 32.5% (29.1%, 30.9% and 39.7% respectively in the three study cohorts). Female gender (p < 0.001), younger age (p = 0.004), higher educational background (p < 0.001), higher annual household income (p = 0.001), private health insurance (p = 0.001) and non-Christian (p < 0.001) were factors associated with more likely CAM use. Multivariate analysis identified female gender (p < 0.001), non-Christian (p = 0.001) and private health insurance (p = 0.015) as independent predictors of CAM use. Most health care professionals thought they did not have adequate knowledge (58.8%) nor were up to date with the best evidence (79.2%) on CAM use in oncology. Health care professionals who used CAM were more likely to recommend it to patients (p < 0.001).

**Conclusions:**

This study demonstrates a similarly high prevalence of CAM use among oncology health care professionals, cancer and non cancer patients. Patients are more likely to disclose CAM usage if they are specifically asked. Health care professionals are interested to learn more about various CAM therapies and have poor evidence-based knowledge on specific oncology treatments. There is a need for further training to meet to the escalation of CAM use among patients and to raise awareness of potential benefits and risks associated with these therapies.

## Background

Complementary and Alternative Medicine (CAM) is 'a comprehensive term used to refer both to traditional medical systems such as traditional Chinese medicine, Indian ayurveda and Arabic unani medicine, and to various forms of indigenous medicine' [1]. The use of CAM has gained enormous popularity among the general public and numerous surveys have reported particularly high prevalence of use in cancer patients [2-5]. Previous studies demonstrated that patients were using CAM without obtaining enough information regarding these therapies [5]. Documented figures of up to 60% of these patients did not disclose their CAM usage to the doctors, and most cited reason was that their doctors did not ask them [4,5]. These findings highlighted the lack of awareness of CAM usage among health care professionals. This could have important oncologic implications due to potential drug-herb-vitamin interactions. For example, shark cartilage has been found to have no effect on tumour growth in clinical trials, but caused severe gastrointestinal toxicity [6,7]. More importantly, St. John's Wort was associated with significantly reduced plasma levels of SN-38, the active metabolite of chemotherapeutic agent, Irinotecan [8]. The majority of novel anticancer treatments are studied in advanced cancer patients and this cohort has been shown to use CAM more frequently [9]. This can be a confounding factor potentially leading to under- or over-estimated drug levels, toxicity, side effect profiles, drug-herb-vitamin interactions and unreliable clinical trial data [10].

Previous surveys in cancer patients have mainly focused on the prevalence and predictors of CAM usage [2-5,9]. In Ireland, the prevalence of CAM use in oncology has been reported in a selected cohort of patients with head and neck cancer [11]. Few studies have assessed the attitudes and perceptions of health care professionals toward CAM use in oncology. Richardson et al reported negative perceptions on CAM by clinical oncologists and Hyodo et al reported discrepant views on CAM between oncologist and cancer patients [12,13]. Risberg et al investigated oncology professionals' knowledge of and attitudes toward CAM in a group of oncologists, nurses, clerks and interventional radiographers [14]. However, the study cohorts did not represent all the members of the current multidisciplinary team. Furthermore, these studies did not assess patients and health care professionals' attitudes and perceptions simultaneously [13,14], which may enable better understanding of the interactions between the two parties. Lastly, health care professionals' knowledge of the use of CAM therapies in specific cancer-related clinical conditions has not previously been investigated.

The aims of this study were therefore to a) investigate the interest and prevalence of CAM use among cancer patients attending a tertiary referral centre in Ireland; b) determine factors associated with CAM usage; (c) assess communications between health care professionals and patients by obtaining opinions and experience from both parties; and (d) investigate health care professionals' knowledge of and attitudes toward CAM.

## Methods

### Participants

This was a single centre cross-sectional survey. Participants were recruited between July and August 2008. Three study cohorts were included in this study, namely cancer patients, non-cancer volunteers and health care professionals. The study was granted approval by the Clinical Research Ethics Committee of the University College Hospital Galway. Patients and volunteers were accrued after explanation of the nature of the survey both in verbal and written format, followed by verbal consent. Cancer patients and non-cancer volunteers were identified from the outpatient clinics, inpatient wards, oncology day ward and radiotherapy department. Cancer patients were patients who have been diagnosed with any cancer in the past. Non-cancer volunteers were patients who were on the wards or attending outpatient clinic for reasons other than cancer, or visitors. Thirty-six consultants across 13 specialties (breast and endocrine surgery; general and gastrointestinal surgery; plastics and reconstructive surgery; urology; head and neck surgery; obstetrics and gynaecology; medical oncology; radiation oncology; dermatology; respiratory medicine; palliative medicine; neurology and haematology) gave permission to have their patients recruited in this study. The study was coordinated by a postgraduate researcher and a medical student undertaking summer research project. Nurses at each study location assisted with the accrual process.

Health care professionals consisted of doctors, nurses, physiotherapists, pharmacists, speech and language, and occupational therapists. All of these health care professionals are involved in the care of cancer patients. Doctors and nurses were asked to complete the questionnaire at various locations within the hospital. For other allied health care professionals, questionnaires were distributed to the corresponding departments.

### Questionnaires

We utilised a modified version of a previously published questionnaire validated in Japan [5]. Prior to the commencement of the survey, the questionnaire was distributed to all participating consultants for review. The structure was further modified and questions reworded according to consultants' feedback. The anonymised questionnaire collected data on sociodemographics; use of CAM and specific details such as types of CAM, expectations and reasons for use; cancer-related characteristics and treatment (additional files 1 and 2). The questionnaire also incorporated Hospital Anxiety and Depression Scale (HADS) which is a validated brief 14-item scoring system to assess emotional state [15]. Data was also collected on the location of consultation, stage of cancer and a simplified Karnofsky performance status score.

The questionnaire distributed to health care professionals was composed of questions regarding their specialties and positions; use of CAM; attitudes towards CAM and previous experience during consultations. Five quiz-like questions regarding the use of CAM in specific cancer-related scenarios were incorporated to determine if health care professionals were up to date with the best available evidence (additional files 1 and 2). The 'correct' answers to these questions were based on level 1a evidence [16-20].

### Statistical Analysis

Statistical analysis was performed using SPSS 15.0 software (Chicago, IL, USA). Univariate comparison of variables was assessed using χ^2 ^test for nominal or ordinal data; Student's t-test and Mann-Whitney U test were used for parametric and non-parametric continuous data respectively. Multivariate analysis was performed using binary logistic regression with forward conditional method. Variables that were significant on univariate analyses were entered into the regression model. A p value of less than 0.05 was considered statistically significant for all tests.

## Results

### Characteristics of Participants

A total of 728 participants were asked to complete the questionnaire, 52 were excluded from subsequent analysis as 5 were erroneously filled out by clerical staff and 47 had excessive missing information. Therefore, 676 questionnaires were valid for analysis including 301 completed by non-cancer volunteers, 219 by cancer patients and 156 by health care professionals. The majority of participants were Caucasians. Nineteen different malignancies were represented in the cancer patient cohort. The prevalence of CAM use among cancer patients, non-cancer volunteers and health care professionals were 29.1%, 30.9% and 39.7% respectively. The prevalence rate in the entire study cohort was 32.5%. Characteristics of cancer patients and non-cancer volunteers are summarised in Table 1, and characteristics of health care professionals are summarised in Table 2.

**Table 1:** Characteristics of patient participants

Variables	Number of Participants	Number of CAM Users (%)	p value (χ2)
Total	520	155 (29.8)	

Gender			<0.001
Male	186	29 (15.6)	
Female	330	124 (37.6)	
Missing	4	2	

Age*	52.5 ± 16.9	49.1 ± 15.5	0.004^†^

Ethnicity			0.385
Caucasian	497	149 (30.0)	
Non-Caucasian	4	2	
Missing	19	4	

Educational background			<0.001
Primary level	97	11 (11.3)	
Secondary level	255	74 (29.0)	
Tertiary level	154	66 (42.9)	
Missing	13	4	

Annual household income			0.001
<€20 000	197	43 (21.8)	
€20 000 - €49 999	161	50 (31.1)	
€50 000 - €99 999	71	33 (46.5)	
>€100 000	12	5 (41.7)	
Missing	79	24	

Health insurance			0.001
None	71 242	26 (36.6) 52 (21.5)	
Public Medical Card			
Private Health Insurance	200	76 (38.0%)	
Missing	7	1	

Religions			0.001
Christian	486	138 (28.4)	
Non-Christian	15	11 (73.3)	
Missing	19	6	

Subgroups			0.369
Non-cancer volunteers	301	93 (30.9)	
Cancer patients	219	62 ((28.3)	
Breast	81	27 (33.3)	0.667
Colorectal	23	4	
Lymphoma	17	6	
Leukaemia	13	3	
Prostate	12	3	
Lung	12	2	
Ovarian	12	5	
Melanoma	12	6	
Head & Neck	7	0	
Oesophagus	5	1	
Kidney	5	1	
Brain	4	1	
Cervix	3	1	
Stomach	3	0	
Testicle	2	0	
Urinary bladder	2	0	
Non-melanoma skin	2	1	
Pancreatic	1	1	
Myeloma	1	0	
Missing	2	0	

HADS			
High anxiety score (≥11)	44	13 (29.5)	0.350
Low anxiety score (<11)	333	112 (33.6)	
Missing	143	30	
High depression score (≥11)	13	3 (23.1)	0.328
Low depression score (<11)	386	128 (33.2)	
Missing	121	24	

Karnofsky score			0.493
80 - 100	106	33 (31.1)	
50 - 70	36	7 (19.4)	
0 - 40	6	1 (16.7)	
Missing	76	24	

* mean ± standard deviation

^† ^student's t-test

**Table 2:** Characteristics of health care professional participants

Variables	Number of Participants	Number of CAM Users (%)	p value (χ2)
Total	156	62 (39.7)	

Gender			0.001
Male	38	7 (18.4)	
Female	118	55 (46.6)	

Age*	31.1 ± 7.3	33.3 ± 8.6	0.001^†^

Ethnicity			0.211
Caucasian	136	56 (41.2)	
Non-Caucasian	18	5	
Missing	1	1	

Professions			0.050
Doctors	59	17 (28.8)	
Nurses	61 27	30 (49.2)	
Physiotherapists	27	10 (37.0)	
Pharmacists	5	4 (80.0)	
Occupational therapists	2	0	
S&L therapists	2	1	

* mean ± standard deviation

^† ^student's t-test

S&L therapists, speech and language therapists

### Types of CAM Use

Biologically-based and orally ingested CAM such as natural supplements (i.e. Probiotics, fish oil, flax seeds, melatonin, etc.), vitamins, green tea and herbal or folk remedies (i.e. garlic, ginger, Essiac, aloe vera, ginseng, Laetrile, etc.) were the most commonly used CAM in the study cohorts. Manipulative and body-based practices such as massage therapy, acupuncture, yoga and chiropractic therapy were popular among CAM users. Energy medicine (i.e. energy healing, biofeedback, etc.), mind-body medicine (i.e. psychotherapy, meditation, etc.) and whole medical systems such as homeopathy and traditional Chinese medicine were less commonly used. The types of CAM used in our study cohorts are summarised in Table 3.

**Table 3:** Types of CAM used

Types of CAM Used	Number of Users (%)
Natural supplements	83 (53.9)

Vitamins	78 (50.6)

Green tea	62 (40.3)

Massage therapy	51 (33.1)

Herbal remedies	50 (30.5)

Acupuncture	40 (26.1)

Yoga	35 (22.7)

Homeopathy	26 (16.9)

Chinese herbal medicine	25 (16.2)

Chiropractic	20 (13.0)

Meditation	15 (9.7)

Energy healing	14 (9.1)

Spiritual practice	13 (8.5)

Music/art therapy	12 (7.8)

Tai Chi	10 (6.5)

Psychotherapy	8 (5.2)

Hypnotherapy	7 (4.5)

Biofeedback	2 (1.3)

Others (Neuro Linguistic Programming)	1 (0.6)

### Predictors of CAM Use

On univariate analysis, female gender (p < 0.001), younger age (p = 0.004), higher educational background (p < 0.001), higher annual household income (p = 0.001), private health care insurance (p = 0.001), non-Christian (p < 0.001) were found to be factors associated with more likely CAM usage. No association was found between ethnicity, HADS and CAM use (Table 1). Multivariate analysis identified female gender (p < 0.001), non-Christian (p = 0.001) and private health care insurance (p = 0.015) as independent predictive factors of CAM use (Table 4).

**Table 4:** Univariate and multivariate analyses of factors predictive of CAM use

	Univariate	Multivariate Binary Logistic Regression		
**Variables**	**p value**	**Likelihood Ratio**	**95% Confidence Interval**	**p value**

Female gender	< 0.001	3.703	2.251-6.094	< 0.001

Younger age	0.004	-		NS

Higher educational background	<0.001	-		NS

Higher annual household income	0.001	-		NS

Private health insurance	0.001	1.670	1.106-2.521	0.015

Non-Christian	<0.001	10.587	3.000-37.359	<0.001

NS, not significant.

In the cancer patient cohort, patients who received hormonal therapy were more likely to use CAM (p = 0.016). Interestingly, no association was found between CAM use and cancer stage, and Karnofsky performance status score.

### Attitudes and Perceptions Toward CAM

Among 155 CAM users, reasons for using CAM were: 72 (51.1%) recommended by family or friends; 42 (29.8%) own will, 12 (8.5%) media influence and 6 (4.3%) recommended by doctor. Among the non-cancer volunteers, CAM users expected CAM to improve immune function (n = 79, 61.7%), general wellbeing (n = 20, 15.7%) and a small proportion expected CAM to prevent cancer (n = 8, 6.3%). On the other hand, the cancer patient cohort used CAM with the expectations that it would cure cancer (n = 1, 0.7%), halt cancer progression (n = 1, 0.7%), improve symptoms (n = 6, 4.1%), and 6 patients used it as a complementary to conventional treatments. When asked if they thought CAM was effective, the majority of CAM users (n = 93, 66.5%) either agreed or strongly agreed. Only 4 participants reported negative effects from CAM use (one constipation and diarrhoea; one drowsiness; one cough, sweating and weight gain; one urinary incontinence).

In 359 non-users, reasons for not using CAM were reported to be: did not have enough information about it (n = 150, 50.2%), no interest (n = 64, 21.4%), did not believe in it (n = 38, 12.7%), never needed it (n = 14, 4.7%), too expensive (n = 12, 4%), happy with conventional medicine (n = 5, 1.7%) and heard bad comments about it (n = 4, 1.3%). Interestingly, 151 (46.6%) of the non-users would like to learn more about CAM.

Among health care professionals, there was a significant association between CAM use and professions (p = 0.050). The prevalence of CAM use was the highest among pharmacist (4/5, 80%), followed by nurses (30/61, 49.2%), physiotherapists (10/27, 37.0%), and the least prevalent among doctors (17/59, 28.8%). Longer duration since qualification was associated with higher likelihood of CAM usage (p = 0.007). There was a high level of interest among health care professionals with 110 (75.3%) wanting to learn more about CAM.

### Communications Between Health Care Professionals and Patients

In 155 CAM users, 43 (30.1%) voluntarily reported CAM use to their doctors. The doctors' response was reported to be: encouraged to continue (n = 16, 37.2%), advised to stop (n = 7, 16.3%), neither discouraged nor encouraged (n = 20, 46.5%), and doctor did not know about CAM (n = 4, 2.8%). Among patients who did not report CAM use voluntarily, only 8 were asked about its use by their doctors. The majority of patients did not mention CAM use because the doctor never asked (n = 47, 34.6%), some thought that the doctor would not understand (n = 5, 3.7%), or would disapprove (n = 8, 5.9%).

From the health care professionals perspective, when asked about their responses to patients regarding CAM use, 26 (17.2%) reported that they would encourage to continue, 5 (3.3%) advise to stop, 92 (60.9%) neither discourage nor encourage. Of these, 58 (38.2%) have been asked about CAM during consultations in the previous 6 months. A large proportion of health care professionals (n = 68, 45.9%) thought they would ask patients about CAM use, while 57 (38.8%) would recommend CAM to patients. Health care professionals who used CAM were more likely to recommend CAM to their patients (p = 0.001).

### Health Care Professionals' Knowledge on CAM

Health care professionals were asked to self-rate their knowledge on CAM. With regards to having adequate knowledge, 1 strongly agreed, 22 (14.4%) agreed, 40 (26.1%) undecided, 62 (40.5%) disagreed and 28 (18.3%) strongly disagreed. When asked if they were up to date with the best available evidence on CAM use, none strongly agreed, 5 agreed, 27 (17.5%) undecided, 83 (53.9%) disagreed and 39 (25.3%) strongly disagreed.

Five questions based on level 1a evidence were designed to assess health care professionals' knowledge on the evidence-based CAM practices including: the role of acupuncture in chemotherapy-induced nausea and vomiting; Chinese herbal medicine for side-effects of chemotherapy; antioxidant for the prevention of lung cancer; oral fish oil for the treatment of cancer cachexia and ginger as an effective anti-emetic remedy. The answers provided are summarised in Table 5. The majority were undecided on all five questions highlighting the lack of knowledge.

**Table 5:** Distribution of answers provided by health care professionals on evidence-based practices of CAM in cancer

	Numbers of Answers (%)				
**Questions**	**Strongly Agree**	**Agree**	**Undecided**	**Disagree**	**Strongly Disagree**

There is evidence that acupuncture is effective in reducing first day vomiting after chemotherapy.	**1 (0.6)**	**19 (12.3)**	128 (82.6)	5 (3.2)	2 (1.3)

There is evidence that Chinese herbs decrease side-effects in patients treated with chemotherapy.	**1 (0.6)**	**11 (7.1)**	132 (85.7)	8 (5.2)	2 (1.3)

There is evidence to support recommending antioxidant vitamins such as α-tocopherol, beta-carotene or retinol to prevent lung cancer.	0	12 (7.8)	127 (82.5)	**9 (5.8)**	**6 (3.9)**

There is evidence to support the use of oral fish oil for the management of cancer cachexia.	0	18 (11.7)	126 (81.8)	**8 (5.2)**	**2 (1.3)**

There is evidence that ginger has a potential role as an antiemetic herbal remedy.	**10 (6.5)**	**29 (18.8)**	109 (70.8)	4 (2.6)	2 (1.3)

Bold fonts indicate the correct answers according to the best available evidence.

## Discussion

In this study, we surveyed the prevalence of CAM use in three distinctive populations and investigated the prevalence, predictive factors, knowledge of and attitudes toward CAM use. The communication on CAM between health care professionals and cancer patients was explored.

One of the strengths of this study is the recruitment process. Participants were approached and invited to complete the questionnaires, instead of using mailed-questionnaire method. With the assistance of nurse coordinators, the response rate of our study was 100% among patients who were invited to participate. This eradicates selection bias inherently associated with most mail-based study design as patients who use CAM are more inclined to participate. One might argue that the prevalence rate of CAM use in this study may not be a true reflection of the entire population as participants were accrued from the hospital setting. However, the prevalence rate reported here is in keeping with previous large scale population surveys [2,21-23]. Furthermore, patients (cancer or non-cancer) and visitors included in this study are a representative group of individuals that hospital-based health care workers interact with on a daily basis and are therefore clinically relevant.

In our study, the prevalence of CAM use is high in all groups of participants, intriguingly, the highest among health care professionals. This would reflect the growing rates of CAM use and it is an encouraging finding as CAM becomes more acceptable in the society. There is no difference in CAM use between cancer patients and non-cancer volunteers, which is not consistent with previous reports [5,22-26]. This may be explained by the inclusion of patients suffering from chronic disorders other than cancer in the non-cancer volunteer cohort. Factors associated with increased CAM use such as female gender, younger age, higher socioeconomic status and private health insurance shown in our study are consistent with previous data [4,5,27,28]. Interestingly, patients with higher anxiety or depression score, more advanced disease stage and poorer performance status are not more likely to use CAM. Kristoffersen et al previously reported higher prevalence of CAM use among cancer patients with poorer prognosis [9]. The authors suggested that this may be due to patients resorting to non-conventional therapy when less hope of cure is given by the physicians. In contrast, other studies have demonstrated that CAM use is not associated with more advanced disease stage [29-31]. This may be related to the complexity of underlying psychological and behavioural mechanisms influencing the use of CAM in cancer patients such as attitude, family support and coping behaviour as have been shown by previous reports [32,33].

The most commonly used CAM is orally ingested agents such as natural supplements, vitamins, green tea and herbal remedies. This further highlights the importance of documentation of the intake of these substances as part of routine clerking and assessment of patients in order to avoid potential drug-herb-vitamin interactions particularly in patients undergoing chemo- or hormonal therapy. As demonstrated by our study, most patients do not inform their doctors about CAM use, mainly because the doctors never ask, or are perceived to be lack of CAM knowledge or disapproving. Therefore the initiatives to elicit CAM usage through history taking may be effective in obtaining such information.

There are numerous reports expressing communication gaps between health care professionals and patients on CAM. This is possibly related to the suboptimal evidence-based knowledge on these therapies but none of these studies had addressed this in detail [4,5,28,34-36]. To our knowledge, this is the first study that includes both health care professionals' attitudes toward CAM use in oncology and an assessment of their knowledge on these therapies. There is a significant discrepancy comparing patients and health care professionals' reported experience toward CAM. While only 8 of 155 CAM users were asked by their doctors about CAM use, a much higher proportion (45.9%) of health care professionals thought they would ask patients about its use. Similarly, only 17.2% of health care professionals would encourage patients to continue CAM, which is markedly different from experience reported by patients that 37.2% of doctors encouraged them to continue CAM when consulted.

When asked about their knowledge on CAM, the majority of health care professionals thought they did not have adequate knowledge (58.8%) nor were up to date with the best available evidence (79.2%) on CAM use. This is evident from answers provided by health care professionals to the five evidence-based CAM questions. Up to 80% were unsure of the roles of the aforementioned CAM practices in cancer-related scenarios, thereby not being able to advise patients regarding the benefits, limitations and even potential harms.

The findings of this study have major implications for undergraduate education. We demonstrated a high prevalence of CAM use in our study population consisting of cancer and non-cancer patients as well as health care workers. Despite the lack of awareness and knowledge on CAM, health care professionals expressed a high level of interest in CAM education. Until recently few allopathic medical students worldwide would have been exposed to the teaching of CAM. In recognition of the growing needs for medical graduates that have at least basic understanding of CAM in order to make appropriate referrals as part of integration of CAM into conventional medicine; several countries have incorporated CAM into undergraduate curricula such as the United States of America (USA), Finland, Germany, Japan, Canada, the Netherlands and Switzerland. Notably in Finland acupuncture has been part of the undergraduate curriculum since 1975. According to the worldwide review on CAM published by the World Health Organization, the majority of medical schools in the USA offer courses on CAM. Since 1997, primary care physicians have been encouraged to attend courses that enable them to incorporate homeopathy into practices. In Germany, medical schools are required to test students' knowledge of CAM. In Australia, acupuncturists form a part of the multidisciplinary management of patients in the public health sector. The British Medical Association recommends incorporating CAM into undergraduate curriculum and making accredited postgraduate training available [37]. University of Southampton commenced education on CAM as part of the Special Study Module (SSM) out of students' request [38]. As part of the Professionalism curriculum, CAM SSM has been made available at the National University of Ireland Galway for the last 2 years. The potential for incorporating CAM as part of a compulsory undergraduate curriculum remains to be evaluated.

Nevertheless there are some limitations to our study. The survey was carried out in a single institution involving generally defined populations of cancer patients, non-cancer volunteers and health care professionals. While further studies may be warranted to investigate the attitudes toward CAM in more specifically defined populations, the present study produced useful information on the overall prevalence of CAM use. The non-cancer volunteer cohort was accrued from the hospital environment, which may not be truly reflective of the general population. Furthermore, the questionnaire used in this study did not address the use of CAM within a specific time period or specifically in relation to cancer.

## Conclusions

This survey demonstrates a high prevalence of CAM use among patients and health care professionals. Increased awareness of CAM use and potential drug-herb-vitamin interactions is critical for optimal patient care in oncology. The incorporation of CAM education into undergraduate medical curriculum may improve health care professionals' knowledge on CAM, thereby improving doctor-patient communication.

## Competing interests

The authors declare that they have no competing interests.

## Authors' contributions

KHC and MAC conceived of and designed the study. KHC performed statistical analysis and drafted the manuscript. RB carried out the questionnaire survey. MAC helped to draft the manuscript. KJS and MJK participated throughout the study and critically reviewed the manuscript. All authors read and approved the final manuscript.

## Pre-publication history

The pre-publication history for this paper can be accessed here:

http://www.biomedcentral.com/1471-2407/11/196/prepub
